# Development and Validation of the Media Health Literacy Scale: Assessment Tool Development Study

**DOI:** 10.2196/62884

**Published:** 2025-05-05

**Authors:** Sangyoon Shin, Seungyeon Kim, Youngshin Song, Hyesun Jeong, Yun Mi Yu, Euni Lee

**Affiliations:** 1 Research Institute of Pharmaceutical Sciences, Natural Products Research Institute College of Pharmacy Seoul National University Seoul Republic of Korea; 2 College of Pharmacy Dankook University Cheonan si, Chungnam Republic of Korea; 3 College of Nursing Chungnam National Univerisity Daejeon si Republic of Korea; 4 Department of Nursing College of Nursing & Health Kongju National University Gongju-si Republic of Korea; 5 Department of Pharmacy and Yonsei Institute of Pharmaceutical Sciences College of Pharmacy Yonsei University Incheon Republic of Korea

**Keywords:** media, internet, media health literacy, ehealth literacy, survey development, validation, health-related information, communication

## Abstract

**Background:**

Advancements in IT have transformed methods for accessing and conveying health-related information. While technical advancements offer more options for people to choose their preferred information sources, injudicious dissemination of incorrect or unverified health-related information by internet-based media poses a threat to society. The concepts of media health literacy (MeHlit) and eHealth literacy have emerged for assessing one’s ability to understand and use health-related information from media sources. However, tools to evaluate the level of MeHlit within the domain of *communication* or follow a solid validation process are scarce.

**Objective:**

This study aimed to develop a validated tool to evaluate the level of MeHlit in adults in South Korea.

**Methods:**

A 2-step tool development process, including item development and validation processes, was carried out. At first, tool development studies were identified by a systematic review of the literature. A conceptual framework was established from the review by constructing an affinity diagram, and an item pool was generated. Face validation was conducted to assess whether the items measured MeHlit properly. Content validation was conducted to assess the overall relationship between domains by calculating the content validity index. Construct validation processes, including exploratory and confirmatory factor analyses, were completed with 1000 adults. Internal consistency of the Media Health Literacy Scale (MHLS) was assessed with Cronbach α. Concurrent validation was conducted to validate the MHLS’s performance by comparing it with an established tool, the Korean version of the eHealth Literacy scale (K-eHEALS).

**Results:**

A total of 13 published studies from the systematic review was used to develop the conceptual framework and an item pool of 65 items was created, including 3 domains (*access*, *critical evaluation*, and *communication*) and 9 subdomains. Through face and content validation processes, the MHLS was refined to comprise 3 domains, 6 subdomains, and 29 items. A total of 1000 participants were recruited for exploratory factor analysis (EFA) and confirmatory factor analysis (CFA). Five subdomains were identified through EFA, and CFA demonstrated a good model fit (chi-square [Cmin *χ*^2^/df] under 2.659, root mean square error of approximation=0.058 [90% CI 0.053-0.062], comparative fit index=0.927, and standard root mean residual under 0.067). Following the EFA and CFA, Cronbach α scores of 0.915 and 0.932, respectively, were obtained, indicating that the tool had good reliability. A positive correlation was found between the MHLS and K-eHEALS from the concurrent validity evaluation, indicating that the MHLS can assess the target concept similarly as the K-eHEALS (Pearson correlation coefficient=0.736, *P*<.001).

**Conclusions:**

The MHLS was developed and validated in a step-by-step process to assess individuals’ ability to access, critically evaluate, and communicate health-related information through media platforms. This validated tool can serve in identifying deficiencies in specific MHLS areas and subsequently providing targeted education.

## Introduction

Advancements in IT have significantly affected how people convey information through various types of media. One of the key changes in transforming communication through the use of new media, including social network services and video-sharing platforms, is a shift from the one-way transmission of information by mass media institutions before the internet to a multidirectional exchange of information [1]. This change in the modes of communication has affected people’s lives as they use health care resources. The multidirectional exchange of health information has empowered patients enabling them to choose services that align with their needs [2]. Although the ease of information delivery has had positive effects, incorrect or unverified information also started to circulate and has led to numerous cases of harm caused by inaccurate health information [3].

The increase of interest in health information on the internet led to the emergence of the concepts of media health literacy (MeHlit) and eHealth literacy. MeHlit was defined as “the ability to identify health-related content across various types of media, recognize its influence on health behavior, critically analyze the content, and express an intention to respond through action” [4]

and eHealth literacy as “the ability to seek, find, understand, and appraise health information from electronic sources and apply the knowledge gained to addressing or solving a health problem” [5].

Several tools have been developed to evaluate media health and eHealth literacy, including a tool to assess MeHlit, the eHealth Literacy Scale (eHEALS), and the eHealth Literacy Questionnaire [5-13]. The translated versions of the eHEALS (K-eHEALS) and the Digital Health Literacy Instrument (Covid-DHL-K) are used in South Korea [14,15]. Previously developed tools include assessment domains that access, understand, and critically appraise information from the internet, or have an intention to change their behaviors; however, they do not provide a communication domain that evaluates the dissemination of health information through the internet. Studies that evaluated internet users’ experiences and behavior through the internet are important in this era of frequent exchange of health information and user opinions; however, not many tools for evaluating the behavior are available, and validated tools are even more scarce.

In the report of the “Survey on the Internet usage” in South Korea, the country’s internet penetration rate was over 99% [16] compared with the global internet penetration rate of 66% [17]. The rate of owning smart devices such as smartphones or tablet PCs is 97.3% [16], and the proportion of people who search for health-related information such as diseases, hospitals, and nutrition through the internet is 60.5% [16]. It can be inferred that the scale of internet accessibility and use in South Korea is significantly greater. Although South Korea is a country with a wide availability of internet access [16] and high literacy rates [18], the rates of health literacy are known to be low [19]. Similar to the global context, problems caused by inaccurate information have also consistently occurred in South Korea [20,21].

Therefore, the objectives of our study are to develop a MeHlit tool, the Media Health Literacy Scale (MHLS), to assess the experiences and behaviors of consumers in accessing, critically evaluating, and communicating health-related information on the internet and to validate the developed tool with responsible processes that can be used in South Korea.

## Methods

### Overview

The study process included the development and validation of a tool to evaluate the MeHlit of Korean adults. The concept of MeHlit was operationally defined as “one’s ability to access, critically evaluate and communicate health-related information by new media,” based on studies related to MeHlit and eHealth literacy [4,22,23]. New media was defined as any channels that people could access and communicate through the internet (eg, social network services, video sharing platforms, etc) using PCs, tablet PCs, smartphones, or any device that can access the internet. Health-related information was defined as any information related to health, such as diseases, medicine, treatment, exercises, nutrients, and other types of information related to health [24]. The tool development was carried out through the processes of item development and validation.

### Conceptual Framework and Item Generation

A systematic literature review was conducted to generate foundational data for the conceptual framework and item pool. Searches using the Medline, Embase, Cochrane Library, KoreaMed, and Research Information Sharing Service databases were conducted to find published studies on tools to evaluate MeHlit and eHealth literacy. Additional search was conducted to identify tools to evaluate media literacy using the same databases, adding Elton B. Stephens Company (EBSCO) and the Web of Science. The keywords used for the literature searches are presented in Multimedia Appendix 1. Studies focusing on the MeHlit, eHealth literacy, and media literacy were eligible for inclusion. Studies that developed an assessment tool to evaluate the literacies and provided a model describing the literacies were also eligible for inclusion. Studies were excluded if they (1) focused on the translation of already developed tools; (2) were not written in either English or Korean; (3) or were not available in full text. The literature search and selection process were performed independently by 2 researchers (SS and SK). Any discordance among the reviewers during the process of literature selection was resolved through mutual agreement or by involving a third researcher (EL) in a discussion.

The tools identified from the systematic review were used to create an appropriate model consistent with the operational definition of MeHlit. An affinity diagram, usually used when various relevant concepts or issues are complex to grasp [25], specified and categorized domains and subdomains within the model; the final conceptual model was constructed by organizing these domains and subdomains [25].

An item pool was created by collecting items identified from the systematic review by listing the tools and items from those tools. Each item was then assigned to the appropriate domain and subdomain within the final conceptual model, with similar items being merged and newly developed items being added. Items presented in English were translated into Korean by 3 internal researchers. The translated items were then modified by internal researchers and a Korean linguist who corrected grammatical errors and ensured cultural adaptation. Each item evaluating MeHlit used a 5-point Likert scale ranging from 1=“strongly disagree” to 5=“strongly agree.”

### Face Validation

Using the initial draft of the survey, face validation was completed with the involvement of 6 internal researchers who have direct experience in interacting with or counseling patients about health-related information. During this process, the appropriateness of each item assignment to a domain or subdomain was reviewed by the researchers based on the objectives of the MHLS. The descriptions of the items and the subdomains were revised to enhance clarity, and redundant items were merged or rearranged to other subdomains after modification, if necessary.

### Content Validation

Content validation was conducted to assess whether items were not only appropriately assigned to the domains and subdomains of the conceptual framework but also relevant to the overall theme of the MHLS, with scores graded by experts [26]. A total of 15 experts (4 medical doctors, 5 nurses, and 6 pharmacists), who met the inclusion criteria of having direct experience in interacting or counseling patients in their practice settings, were recruited to conduct content validation.

The expert panel provided feedback on the tool in general, domains, subdomains, and items by rating their level of agreement on a scale of 1 to 4, where 1 indicated that items were not related to domains and subdomains, 2 that they were slightly related, 3 that they were related, and 4 that they were highly related. The scoring was then converted, with scores of 1 and 2 coded as 0, implying disagreement, and scores of 3 and 4 coded as 1, implying agreement that items were allocated properly. The scores for each item were summed and divided by the number of participating experts (n=15) to calculate the content validity index (CVI), based on which items with a CVI score of 0.8 or higher were retained; those with a score between 0.7 and 0.8 required further modification, enhancement, or deletion; and those with a score below 0.7 were removed from the item pool [26].

### Pilot Test

A pilot test was conducted with general consumers by snowball sampling. A total of 30 participants representing general consumers who were not health care professionals completed the revised MHLS draft generated from the content validation and provided comments for further revision.

### Construct Validation

A survey was conducted for construct validation using the MHLS semifinalized draft prepared based on the content validation and the pilot testing. The process included descriptive analysis, item analysis, factor analysis, and reliability tests. Sample size calculation was based on the literature indicating the appropriate number of participants for conducting factor analysis. The number of participants over 500 was considered very good [27,28]; the ratio of the number of participants and the number of survey items would be sufficient as 10:1 [29]; the number of participants should be equal or exceed 20 times more than the number of factors [27]. Based on the calculation, the study determined that 500 participants in each exploratory and confirmatory factor analysis (CFA) were necessary [30]. A total of 1000 participants aged 19 and above were recruited for each exploratory factor analysis (EFA) and confirmatory factor analysis. The survey participants were eligible if they met the inclusion criteria of having searched for health-related information on the internet or media and having delivered health-related information through media platforms. Health care professionals were excluded because they were considered familiar with health-related information due to their training in looking for and evaluating such information. A stratified sampling scheme was used to reflect the sex and age corresponding to the population distribution in South Korea [31]. The survey was conducted between December 14 and November 22, 2021 [31].

Item analysis was conducted to evaluate the quality of each item. The mean, skewness, and kurtoses of each item and the item-total correlation were calculated. Items with skewness and kurtosis values outside the range of ± 1 were excluded [32]. Regarding item-total correlation, an item with a correlation value of less than 0.3 indicated a low correlation and was excluded [33].

The Kaiser-Meyer-Olkin (KMO) measure and Bartlett sphericity test were used to assess the suitability of the respondent data for factor analysis [28,34]. Following the EFA results, eigenvalues, variance percentages, and factor loadings of items were identified. Items with factor loadings below 0.40 were removed. All items were then reviewed to determine whether they were grouped with similar items in a factor and those that were considered to have different characteristics from other items in the factor were eliminated after discussion with the researchers [28]. The process of EFA was conducted with SPSS (version 26; IBM Corp) [35].

The model provided by the EFA was validated through CFA. Model fit was assessed based on chi-square tests, root mean square error of approximation (RMSEA), comparative fit index (CFI), and the standardized root mean residual (SRMR). The model was considered to have a good fit when each index satisfied the following scores: RMSEA under 0.5, CFI over 0.95, and SRMR under 0.08 [34]. The internal consistency reliability was tested by calculating the Cronbach α value. A Cronbach α value of 0.7 or higher indicated acceptable internal consistency and this tool was determined to be reliable [36]. The process of CFA was conducted with SPSS Amos (version 26; IBM Corp) [37].

### Concurrent Validation With the K-eHEALS

The MHLS tool was validated using the Korean version of the eHealth Literacy scale (K-eHEALS) to conduct concurrent validation, which aims to confirm the construct validity of the MHLS by comparing it with previously developed tools. This process involved examining the correlation between the K-eHEALS, which was a translated version of eHEALS) and had similar evaluation criteria, and the MHLS tool developed in this study, using Pearson correlation coefficients [38]. Since the K-eHEALS did not include items corresponding to the Communication domain of the MHLS, the comparison was conducted using the K-eHEALS and MHLS scores, excluding the *communication* domain. The process of calculating a correlation score was conducted with SPSS (version 26; IBM Corp) [35].

### Ethical Considerations

The study protocol adhered to an appropriate ethical review and was approved by the Institutional Review Board of Seoul National University (2108/002-01). The study conformed to the principles embodied in the Declaration of Helsinki. The participants were recruited through the Tillion Internet research panel managed by a survey company, Panel Marketing Interactive (PMI Co, Ltd). A web-based survey was accessed by the participants through the website of the company. Before participating in the study, the participants were informed of the purpose, procedures, data use, and their rights. Then the participants signed a consent form stating that their participation was voluntary and they could withdraw at any point. As data protection measures, the collected data from the web-based survey were anonymized and no personal identifiers were collected. Participants who completed the survey received a reward in the form of company points redeemable on the web-based platform.

## Results

### Draft of the MHLS

A systematic review searching media literacy, MeHlit, and eHealth literacy found literature that suggested models of the literacies. The results showed 13 models of related literacies [9-12,23,39-45]. Through the affinity diagram, domains of models were grouped with domains that present similar meanings. From the results of the affinity diagram, the European Association for Viewers Interests (EAVI) model, which included the domains of *use*, *critical understanding*, and *communicative abilities*, was selected as the main framework because of its comprehensive characteristics and similarity with operational definition. All domains from collected models were regrouped under these 3 domains of the EAVI model (Multimedia Appendix 2). The conceptual framework to develop survey items was modified and constructed based on the result of the affinity diagram, resulting in 3 domains (*access*, *critical evaluation*, and *communication*) and 9 subdomains (Table 1). The item pool was constructed using the collected questionnaires (65 items).

**Table 1:** Change in domains, subdomains, and number of items.

	MHLS^a^ draft			After face validation			After content validation			After construct validation	
Domains	Subdomains	Items	Subdomains		Items	Subdomains		Items	Subdomains		Items
Access	3	17	3		12	3		11	2		10
Critical evaluation	3	29	1		11	1		10	1		9
Communication	3	19	2		10	2		8	2		8
Total	9	65	6		33	6		29	5		27

^a^MHLS: Media Health Literacy Scale.

Face and content validation were conducted qualitatively. During face validation, the domains, subdomains, and all items in the conceptual framework were reviewed. As a result, the number of subdomains decreased from 9 to 6 and the number of items decreased from 65 to 33 (Table 1). Content validation resulted in a reduction of 4 items, considering the relationship between items and domains. One item with a CVI score higher than 0.8 was deleted because of duplication, and another with a CVI score between 0.8 and 0.7 was deleted because of a poor relationship with MeHlit. A total of 6 items with a CVI score under 0.7 should have been deleted, but 2 items, each within the “evaluation of quality on health-related information” subdomain of the *critical evaluation* domain and the “experience of communicating health-related information” subdomain of the *communication* domain were modified and retained because of their importance as judged by internal researchers. The scale-level CVI score based on the average method was 0.83 and the scale-level CVI score based on universal agreement method was 0.88.

In the pilot test, 30 general consumers were recruited through snowball sampling. Their comments from the process were used to modify the MHLS draft. These comments were related to the operational definition of health-related information and the mode of accessing the internet, such as through PC or mobile. Therefore, a descriptive statement on the meaning of health-related information in our study was added to the MHLS in a box format. We also elaborated on the description that various modes of accessing the Internet were acceptable (Multimedia Appendix 3).

### Construct Validation of the MHLS

Following content validation of the MHLS draft, item analysis, factor analysis, and reliability test were conducted to achieve construct validity (Multimedia Appendix 4). A total of 1000 participants answered the MHLS items after content validation and were divided into 2 groups: 498 in the EFA and 502 in the CFA. The mean (SD) age of the participants was 42.49 (SD 12.37) years in the EFA and 42.58 (SD 12.31) years in the CFA. Male participants comprised 271 of 498 participants in the EFA and 273 of 502 in the CFA (54.4%) and college graduates or higher comprised 416 of 498 participants in the EFA and 421 of 502 in the CFA (83.7%). A total of 381 of 498 participants in the EFA and 402 of 502 in the CFA were employed (78.2%). The characteristics of participants are summarized in Table 2.

Item analysis revealed a mean of 3.20 to 4.32. The skewness and kurtosis of whole items were in the range of ± 1, and the item-total correlation of whole items was higher than 0.3 (Multimedia Appendix 5).

The KMO index was 0.938, and Bartlett sphericity showed a statistically significant result (*P*<.001), with both indices indicating the EFA was appropriate. The EFA identified 5 factors that explained 52.726% of cumulative variance. Direct oblimin rotation was used and 2 items were deleted considering the factor loading on each factor. One was excluded because the factor loading was under 0.4; the other one about the experience with accessing health-related information via media was excluded because the factor loading was over 0.4 with the utilization of digital devices. Factors from EFA were named considering items that described each factor: “digital device operational skills,” “experience and ability of accessing health-related information,” “evaluation of quality of health-related information,” “experience of communicating health-related information,” and “responsible communication of health-related information.” The EFA identified 5 subdomains and 27 items (Table 3).

**Table 2:** Characteristics of participants in construct validation.

Characteristics					Exploratory factor analysis (n=498), n (%)		Confirmatory factor analysis (n=502), n (%)
**Age (years)**							
		19-29		103 (20.7)		104 (20.7)	
		30-39		117 (23.5)		117 (23.3)	
		40-49		125 (25.1)		126 (25.1)	
			50-59	104 (20.9)		104 (20.7)	
			60	49 (9.8)		51 (10.2)	
**Sex**							
		Male		271 (54.4)		273 (54.4)	
		Female		227 (45.6)		229 (45.6)	
**Employment status**							
	Yes				381 (76.5)		402 (80.1)
	No				117 (23.5)		100 (19.9)
**Education level**							
	Middle school graduate				1 (0.2)		1 (0.2)
	High school graduate				81 (16.3)		80 (15.9)
	College graduate or higher				416 (83.5)		421 (83.9)
**Average monthly income**							
			5th quintile	53 (10.6)		45 (9.0)	
			4th quintile	75 (15.1)		77 (15.3)	
			3rd quintile	128 (25.7)		134 (26.7)	
			2nd quintile	141 (28.3)		144 (28.7)	
			1st quintile	101 (20.3)		102 (20.3)	

**Table 3:** Exploratory factor analysis of the Media Health Literacy Scale.

Subdomains and items		Factor loadings				
		1	2	3	4	5
**Digital device^a^ operational skills**						
	I can access the internet via a digital device.			–.819		
	I can install programs or applications on digital devices.			–.732		
	I can operate a digital device to navigate a search engine.			–.754		
	I can operate a digital device to communicate or post information.			–.697		
**Experience and ability to access health-related information^b^**						
	When I come across health-related information, I browse the internet for additional related information.				.519	
	When I want to get health-related information, I generally browse the websites of health-related public institutions or hospitals.				.498	
	I have experience finding desired health-related information on the internet.				.446	
	I know where to search for health-related information on the Internet.				.627	
	I know which search terms to use to find health-related information on the internet.				.592	
	I can choose the desired information from the abundant pool of health-related information available on the internet.				.500	
**Evaluation of quality on health-related information**						
	I consider whether the health-related information I come across on the internet is accurate.	.454				
	I check whether the health-related information I come across on the internet is up to date.	.586				
	I check the sources of the health-related information I come across on the internet.	.683				
	I check whether the health-related information I come across on the internet is provided by health care professionals^c^.	.613				
	I check whether the health-related information I come across on the internet is corroborated by scientific evidence^d^.	.603				
	I check for intentions or purposes (eg, political leaning or advertising) behind the health-related information I come across on the internet.	.561				
	I check whether the health-related information I come across on the internet highlights only the positive effects or conceals the risks.	.606				
	I check the accuracy of the health-related information I come across on the internet by cross-referencing multiple websites.	.520				
	I check the accuracy of the health-related information I come across on the internet by consulting a healthcare professional.	.609				
**Experience in communicating health-related information**						
	I press the “recommend”, “do not recommend”, “like”, or “dislike” buttons on health-related information posts I come across on the internet to express my opinion^e^.		.723			
	I write comments on health-related information posts I come across on the internet to express my opinion.		.817			
	I forward health-related information posts I come across on the internet to others via messaging apps.		.676			
	I post texts, images, or videos with health-related information on the internet (eg, blog, online forum, YouTube, social media).		.710			
**Responsible communication of health-related information**						
	I check whether the health-related information on the internet is accurate when I post, forward, or express opinions through the internet.					.585
	I believe that the health-related information that I post, forward, or comment on over the internet (including likes, recommendations, and comments) may affect the health or well-being of others or society.					.561
	I check whether the health-related information I post, forward, or comment on over the internet (including likes, recommendations, and comments) contains any material that violates the law.					.520
	I disclose the source of the online health-related information when I post or forward it.					.499
Eigenvalue		8.76	4.26	1.47	1.20	0.86
Proportion of variance (total 52.726%)		30.47	14.38	3.67	2.73	1.48

^a^Digital device: smartphone, computer, and tablet PCs.

^b^Health-related information: news, advertisements, articles, blogs, content on YouTube, and other social media.

^c^Health care professionals: physicians, doctors of Korean medicine, dentists, pharmacists, nurses, nursing assistants, and so on.

^d^Scientific evidence: evidence provided by health care professionals, professional organizations, journal articles, government documents, and so on.

^e^Opinions: likes, recommendations, and comments.

**Figure 1:**
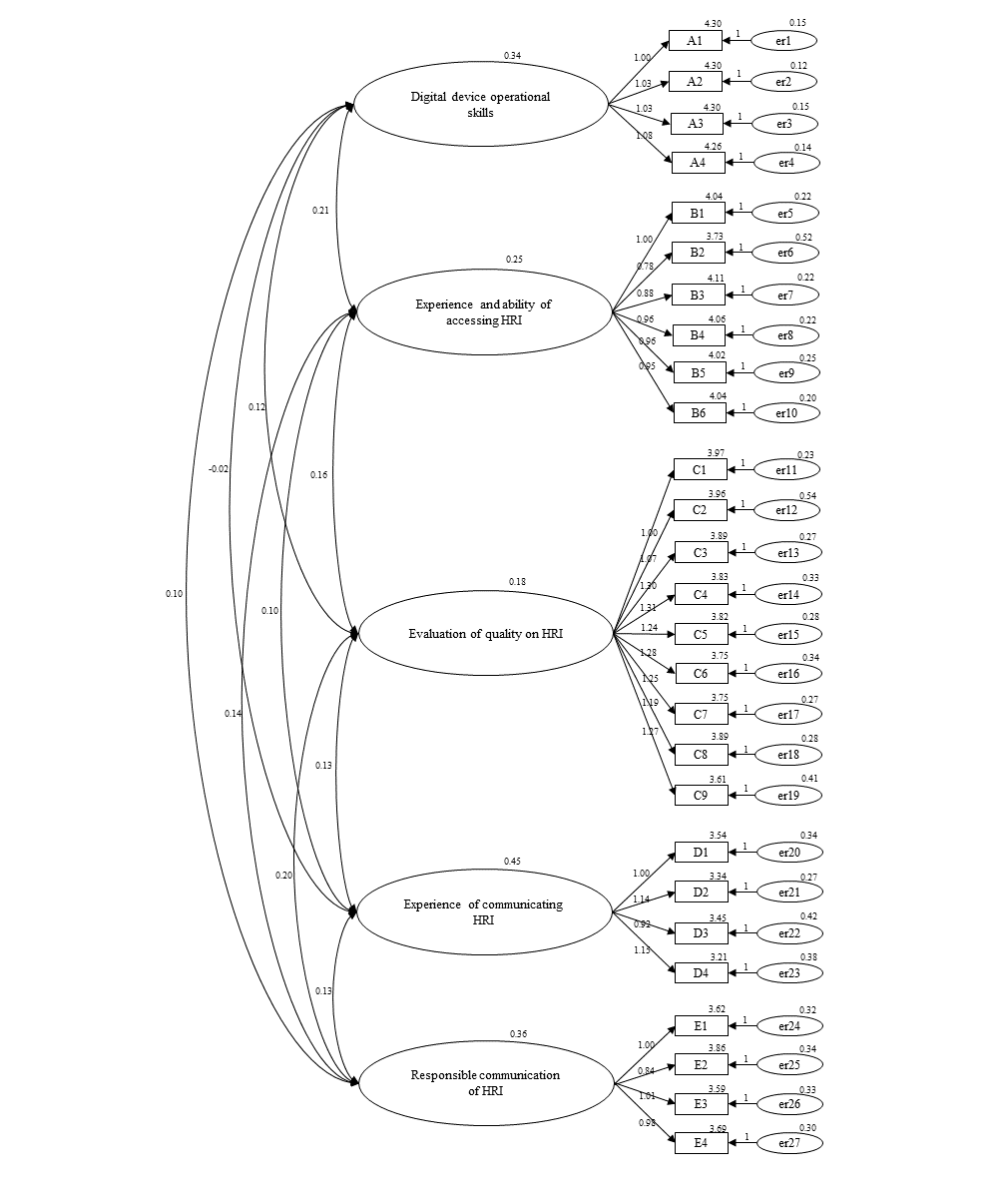
Structural equation model for confirmatory factor analysis. HRI: health-related information.

CFA was conducted with a 5 factors model derived from the EFA. In total, 502 sex- and age-stratified samples from 1000 participants were included in the CFA. The CFA results were presented as: Cmin *χ*^2^ (*df*) under=2.659, RMSEA=0.058 (90% CI 0.053-0.062), CFI=0.927, and SRMR under 0.067. All indicators represented a good fit for the model. The structural equation model is presented in Figure 1.

Internal consistency was confirmed with Cronbach α. The Cronbach α score of the dataset used for EFA was 0.915, and the domain scores were as follows: “digital device operational skills”=0.888, “experience and ability of accessing health-related information”=0.826, “evaluation of quality on health-related information”=0.870, “experience of communicating health-related information”=0.864, and “responsible communication of health-related information”=0.748. The Cronbach α score of the dataset used for CFA was 0.927, and the domain scores were as follows: “digital device operational skills”=0.911, “experience and ability of accessing health-related information”=0.824, “evaluation of quality on health-related information”=0.888, “experience of communicating health-related information”=0.849, and “responsible communication of health-related information”=0.804. Scores indicated acceptable internal consistency.

### Concurrent Validation of the MHLS

The correlation between the K-eHEALS and MHLS scores, excluding the *communication* domain, had a positive relationship. This indicates that the MHLS can measure the target concept similar to the K-eHEALS. The Pearson correlation coefficient was 0.736, showing statistical significance (*P*<.001).

## Discussion

### Principal Findings

Increased health concerns and interests in health-related information [46] have resulted in a proliferation of related information in the media, which has affected public behavior [47,48]. Although various tools have been developed to evaluate how people interact with health-related information in the media [5-13,45], there has been no tool to comprehensively assess the concepts following a rigorous validation process. The MHLS was developed to address this gap by offering quantitative scores measuring MeHlit across the domains of *access*, *critical evaluation*, and *communication*. We believe the MHLS tool can identify consumers with relatively low scores in specific domains and the potential to cause harm by disseminating misinformation, disinformation, or malinformation.

The development process was based on a framework that proposed these 3 abovementioned domains. The MHLS, structured by *access*, *critical evaluation*, and *communication*, can measure the proficiency of individuals who interacted with health-related information via media. In the study by Nazarnia et al [8], the MeHLit tool was heavily focused on the capability of users to appraise health-related information with the domains of *goal appraisal*, *content appraisal*, *implicit meaning appraisal*, *visual comprehension*, and *audience appraisal skill*. In addition, the eHEALS, consisting of 6 domains covering traditional, health, information, scientific, media, and computer literacy [5], has been used in several studies to assess eHealth literacy [49-51]. Surveys like the Digital Health Literacy Instrument, eHealth Literacy Questionnaire, and eHEALS surveys address users’ capability without collecting their experience of using the internet. Levin-Zamir et al [6] and Fleary [7] also developed tools to evaluate MeHlit with the domains of *identification or recognition*, *influence or critical analysis*, and *action or reaction*. While these tools evaluated the user’s intention to act or react, the intention-behavior gap has been documented in the literature [52], tools evaluating not only consumers’ intentions but also their behaviors are needed.

The main difference between the preexisting tools and the MHLS tool in our study is the inclusion of the *communication* domain, which elucidates how individuals interact with health-related information. We believe the MHLS tool distinguishes itself from already developed tools by revealing consumers’ behavior on communicating health-related information they have searched for and their opinions, allowing assessment of both the transmission of health-related information and individuals’ responsibility in health-related information dissemination. In addition, where harm by the dissemination of misinformation is prominently evident [3,53], the tool can grasp the characteristics of people who have shared misinformation without realizing it and identify weaknesses to improve prevention of harm caused by misinformation.

The MHLS demonstrated structural strengths through well-executed development and validation processes. During the development stage, a systematic review of relevant literature was conducted to establish the conceptual framework and formulate items. Since there are various ways to develop items and a conceptual framework, [54,55] appropriate methods were chosen based on the conditions of the research environment. The drafted items underwent a series of validation processes to ensure qualitative relationships with the items recommended in the literature [56]. Construct validation, including factor analysis (both exploratory and confirmatory), and reliability and concurrent validity tests were conducted to quantitatively validate the tool [34,36,38]. Compared with the already developed tools, the EFA scores were demonstrated with KMO, which identifies whether an appropriate number of participants were recruited. EAVI scores were 0.859, 0.896, and 0.93 and the MHLS scored 0.938, meaning the number of participants included was very good [8-10]. CFA scores were presented as CFI and RMSEA, which explains the fitness of the final model. Previous studies reported CFI values of 0.93 and 0.94 and RMSEA values of 0.049, 0.051, and 0.07, which represent a good fit of the model [8,9,13], while the MHLS had CFI and RMSEA scores of 0.927 and 0.058. The reliability of the already developed tools according the Cronbach α scores ranged from 0.74 to 0.93 [5,6,8-10] and the Cronbach α of the MHLS was 0.915 in the EFA and 0.927 in the CFA, indicating good reliability. In comparison with already developed tools, all validation steps of the MHLS scored similarly to the past tools, satisfying the criteria for being good. This quantitative comparison between already developed tools shows that meticulous development and validation procedures support the validity of the MHLS [57].

The EFA results recommended that the model include factors with eigenvalues greater than 1.0, but our model included a factor with an eigenvalue less than 1.0 because the model demonstrated a stronger relationship between items and factors based on factor loadings and item allocation [58]. Subsequently, the CFA results indicated that the model had a good fit, surpassing the acceptable criteria. Structural equation modeling provided additional support for the model’s strong fit. The model effectively explained MeHlit, beginning with the construction of a framework and ensuring several types of validity.

To further confirm the validity of the MHLS, our study conducted concurrent validation with the K-eHEALS [38]. The eHEALS was originally developed to assess eHealth literacy among individuals using IT, while the K-eHEALS is the translated version specifically designed for Korean adults [5,14]. As eHealth literacy shares several similarities with MeHlit, analyzing the correlation between the K-eHEALS and the MHLS can validate the effectiveness of the MHLS based on the correlation coefficient scores. The results indicated a positive correlation between the tools, suggesting that the MHLS effectively measures the level of MeHlit among survey participants.

### Limitations

There are several limitations in our study. First, generalization of the MHLS can be challenging because the characteristics of participants did not represent those of the general public. The proportion of college graduates or higher in our study was about 84%, which is significantly higher than the national statistics of 53% [59]. Moreover, because the panel used in the study recruited participants by email or over the internet, those who were not familiar with the recruiting method were not included. Therefore, only 10% of participants were aged 60 years or more, compared with approximately 14.7% in the Korean population [60]. Participants older than 70 years and more were not represented in this study. Since the reported rate of searching the internet among people 70 years and older was more than 50% in South Korea and there was a high representation of people with college-level or greater education, the exclusion of these populations indicates that the study population may not ideally represent the general public in South Korea. Therefore, further studies to ensure the validity of the MHLS are needed, especially targeting older adults and consumers with low educational levels.

Second, the MHLS tool was a home-grown tool for consumers in South Korea. Therefore, the application of the MHLS to other cultural environments should consider cultural and linguistic differences. We believe cultural and societal characteristics, including the rate of internet penetration, health information–seeking behaviors, education levels, literacy or health literacy rates, health policy, health care access, and service products could vary across cultures; they should be considered when translating the MHLS for other cultures and should go through additional validation processes.

### Conclusions

The MHLS was developed and validated in a step-by-step process to assess individuals’ ability to access, critically evaluate, and communicate health-related information through media platforms. The findings from our study suggest that the validated MHLS tool performed well at measuring MeHlit in South Korea.

## Appendix

Multimedia Appendix 1 Search keywords.

Multimedia Appendix 2 Affinity diagram.

Multimedia Appendix 3 Media Health Literacy Scale.

Multimedia Appendix 4 Checklist for Reporting Results of Internet E-Surveys (CHERRIES) checklist.

Multimedia Appendix 5 Item analysis results.

## Abbreviations

CFA: confirmatory factor analysis
CFI: comparative fit index
CVI: content validity index
EAVI: European Association for Viewers Interests
EBSCO: Elton B. Stephens Company
EFA: exploratory factor analysis
eHEALS: eHealth Literacy Scale
K-eHEALS: the Korean version of the eHealth Literacy scale
KMO: Kaiser-Meyer-Olkin
MeHlit: media health literacy
MHLS: Media Health Literacy Scale
RMSEA: root mean square error of approximation
SRMR: standardized root mean residual

## Data Availability

The datasets generated or analyzed during this study are available from the corresponding author on reasonable request.
