# Supplementary material for: Development and Validation of the Media Health Literacy Scale: Assessment Tool Development Study
Source: J Med Internet Res. 2025 May 5;27:e62884. doi: 10.2196/62884 (PMC12089881; doi:10.2196/62884)
Supplement: Multimedia Appendix 1 [file jmir_v27i1e62884_app1.docx]

Multimedia Appendix 1. – Search keyword

| **Media Literacy** | | **Boolean operator** |
| --- | --- | --- |
| Media | (Media Literacy) or (Media Evaluation) or (Media Education) or (Media Campaign) | And |
| Measurement | (Measure) or (Scale) or (Tool) or (Assess) or (Level) or (Test) or (Develop) or (Valid) or (Quantify) or (Questionnaire) or (Survey) or (Analysis) or (Evaluate) | And |
| Literacy | literac* |  |
| **Media Health Literacy** | |  |
| Media Health | “media health” | And |
| Literacy | literac* |  |
| **eHealth Literacy** | |  |
| eHealth | (ehealth) or (e-health) or (mhealth) or (m-health) or (mobile health) or (digital health) or (internet health) or (online health) or (internet-based health) or (computer health) or (computer-based health) or (web health) or (web-based health) | And |
| Literacy | literac* |  |

2. Media Health Literacy

3. eHealth Literacy
