# Supplementary figures and images for: Development and Validation of the Media Health Literacy Scale: Assessment Tool Development Study
Source: J Med Internet Res. 2025 May 5;27:e62884. doi: 10.2196/62884 (PMC12089881; doi:10.2196/62884)

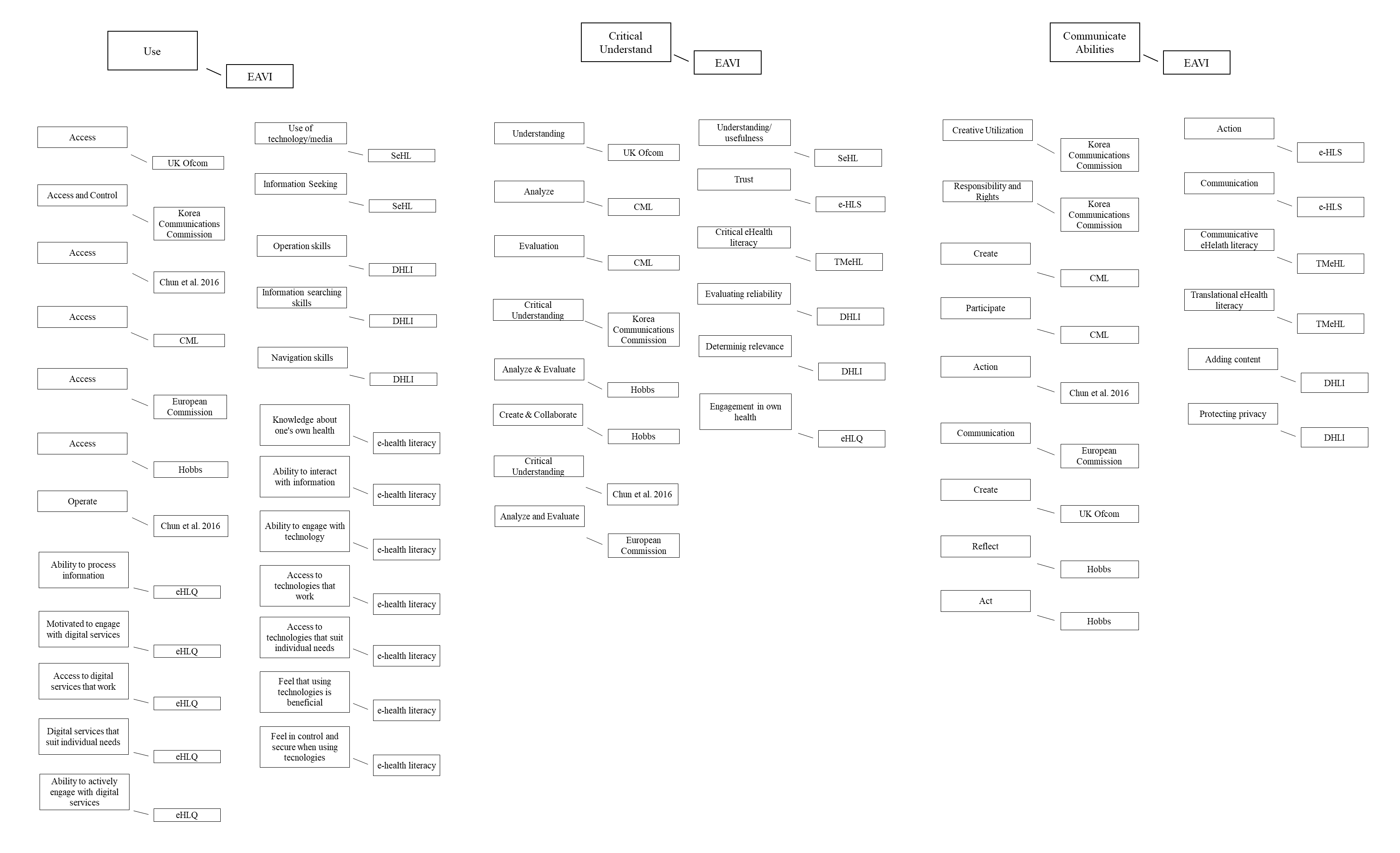

Supplement: Multimedia Appendix 2 [file jmir_v27i1e62884_app2.png]
