# Supplementary material for: Development and Validation of the Media Health Literacy Scale: Assessment Tool Development Study
Source: J Med Internet Res. 2025 May 5;27:e62884. doi: 10.2196/62884 (PMC12089881; doi:10.2196/62884)
Supplement: Multimedia Appendix 3 [file jmir_v27i1e62884_app3.docx]

# Media Health Literacy Scale

**(MHLS)**

**This survey aims to assess the “ability to access, critically evaluate, and communicate health-related information using the media” among Korean adults.**

**There are no correct or incorrect answers to the questions in this survey.**

**The term, “health-related information” refers to all information related to health, such as information on diseases, medication, food, and exercise.**

**The content and results of the survey will never be used for any purpose other than research.**

**Screening**

SQ1. How old are you? _____ years old

SQ2. What is your current occupation?

1. Self-employed (up to 9 employees)
2. Sole proprietorship (10 or more employees)
3. Sales/service (salesperson, clerk, etc.)
4. Technical/production worker (factory worker, driver, etc.)
5. Managerial/administrative work (department manager level or higher, civil servant of level 4 or higher, etc.)
6. Clerical work (office worker below the deputy manager level, civil servant of level 5 or lower, etc.)
7. Healthcare professional (physician, pharmacist, nurse, etc.)
8. Specialized professional (engineer, professor, lawyer, journalist, etc.)
9. Teacher, private academy instructor
10. Worker in the agriculture/forestry/fishing/livestock industry
11. Undergraduate/graduate student
12. Homemaker
13. Unemployed

9997) Other (Please specify: )

**“Health-related information” refers to any information related to health, such as information on diseases, medication, food, and exercise.**

SQ3. Have you ever searched for HRI on the Internet using smartphones, computers, tablet PCs, etc.?

1. Yes
2. No

SQ4. Have you ever used the Internet to post, forward, or comment on (including likes, recommendations, comments, etc.) any HRI you found online?

1. Yes
2. No

**Health-Related Questions**

A1. How do you rate your health?

1. Very poor
2. Poor
3. Average
4. Good
5. Excellent

A2. Have you ever been diagnosed with any chronic disease?

(e.g., diabetes, hypertension, hyperlipidemia, asthma, rhinitis, atopy, allergies, stroke, lung disease, liver disease, arthritis, epilepsy, cancer)

## Yes

1. No

A3. How interested are you in your health?

1. Not at all interested
2. Not interested
3. Neither uninterested nor interested
4. Interested
5. Very interested

**Internet Usage Questions**

B1. How often do you use the Internet?

1. More than once a day (_____ hours a day on average)
2. At least once a week (_____ hours per week on average)
3. At least once a month (_____ hours per month on average)
4. Less than once a month (_____ hours per year on average)

B2. How often do you use the Internet to search for HRI?

1. More than once a day (_____ hours and _____ minutes per day on average)
2. At least once a week (_____ hours and _____ minutes per week on average)
3. At least once a month (_____ hours and _____ minutes per month on average)
4. Less than once a month (_____ hours and _____ minutes per year on average)

B3. Please select all the media you use to search for HRI on the Internet.

1. Internet news or articles
2. Search engines (e.g., Naver, Daum, Google)
3. Video services (e.g., YouTube, Naver TV, KakaoTV, AfreecaTV)
4. Social media (e.g., blogs, online forums, Facebook, Instagram, Naver Band, X, KakaoStory, podcasts)
5. Messengers (e.g., KakaoTalk, Facebook Messenger, Line)

9997) Other (specify: )

B4. According to you, how trustworthy is the HRI available online?

1. Not at all trustworthy
2. Not trustworthy
3. Neither untrustworthy nor trustworthy
4. Trustworthy
5. Very trustworthy

B5. How much do you think you need the HRI available online?

1. Not at all needed
2. Not needed
3. Unsure
4. Needed
5. Very much needed

B6. How useful do you find the Internet when making health-related decisions?

1. Not at all useful
2. Not useful
3. Unsure
4. Useful
5. Very useful

B7. According to you, how important it is to have access to online HRI?

1. Not at all important
2. Not important
3. Unsure
4. Important
5. Very important

**Media Health Literacy Questions**

**The following questions are regarding the ability to use digital devices (smartphones, computers, tablet PCs, etc.). Please rate your ability to perform the following according to the degree to which you agree with each statement.**

C1. I can access the Internet via a digital device (e.g., smartphone, computer, tablet PCs).

1. Strongly disagree
2. Disagree
3. Neither agree nor disagree
4. Agree
5. Strongly agree

C2. I can install programs or applications on digital devices (e.g., smartphones, computers, tablet PCs).

1. Strongly disagree
2. Disagree
3. Neither agree nor disagree
4. Agree
5. Strongly agree

C3. I can operate a digital device (e.g., smartphone, computer, tablet PC) to navigate a search engine.

1. Strongly disagree
2. Disagree
3. Neither agree nor disagree
4. Agree
5. Strongly agree

C4. I can operate a digital device (e.g., smartphone, computer, tablet PC) to communicate or post information.

1. Strongly disagree
2. Disagree
3. Neither agree nor disagree
4. Agree
5. Strongly agree

**The following questions are regarding your experience and ability of browsing the Internet for “health-related information.” Please rate your experience on the following according to the degree to which you agree with each statement.**

**“Health-related information” refers to any information related to health, such as information regarding diseases, medication, food, and exercise.**

D1. When I come across HRI (e.g., news, advertisements, articles, blogs, content on YouTube and other social media), I browse the Internet for additional related information.

1. Strongly disagree
2. Disagree
3. Neither agree nor disagree
4. Agree
5. Strongly agree

D2. When I want to get HRI, I generally browse the websites of health-related public institutions or hospitals.

1. Strongly disagree
2. Disagree
3. Neither agree nor disagree
4. Agree
5. Strongly agree

D3. I have experience finding desired HRI on the Internet.

1. Strongly disagree
2. Disagree
3. Neither agree nor disagree
4. Agree
5. Strongly agree

D4. I know where to search for HRI on the Internet.

1. Strongly disagree
2. Disagree
3. Neither agree nor disagree
4. Agree
5. Strongly agree

D5. I know which search terms to use to find HRI on the Internet.

1. Strongly disagree
2. Disagree
3. Neither agree nor disagree
4. Agree
5. Strongly agree

D6. I can choose the desired information from the abundant pool of HRI available on the Internet.

1. Strongly disagree
2. Disagree
3. Neither agree nor disagree
4. Agree
5. Strongly agree

**The following questions are about your ability and behavior regarding assessing “health-related information” you come across on the Internet. Please rate how often you engage in the following activities.**

**“Health-related information” refers to any information related to health, such as information regarding diseases, medication, food, and exercise.**

E1. I consider whether the HRI I come across on the Internet is accurate.

1. Strongly disagree
2. Disagree
3. Neither agree nor disagree
4. Agree
5. Strongly agree

E2. I check whether the HRI I come across on the Internet is up to date.

1. Strongly disagree
2. Disagree
3. Neither agree nor disagree
4. Agree
5. Strongly agree

E3. I check the sources of the HRI I come across on the Internet.

1. Strongly disagree
2. Disagree
3. Neither agree nor disagree
4. Agree
5. Strongly agree

E4. I check whether the HRI I come across on the Internet is provided by healthcare professionals.

* Healthcare professionals: physicians, doctors of Korean medicine, dentists, pharmacists, nurses, nursing assistants, etc.

1. Strongly disagree
2. Disagree
3. Neither agree nor disagree
4. Agree
5. Strongly agree

E5. I check whether the HRI I come across on the Internet is corroborated by scientific evidence.

(Examples of scientific evidence: opinions of healthcare professionals, opinions of specialized agencies, research articles, reports, etc.)

1. Strongly disagree
2. Disagree
3. Neither agree nor disagree
4. Agree
5. Strongly agree

E6. I check for intentions or purposes (e.g., political leaning or advertising) behind the HRI I come across on the Internet.

1. Strongly disagree
2. Disagree
3. Neither agree nor disagree
4. Agree
5. Strongly agree

E7. I check whether the HRI I come across on the Internet highlights only the positive effects or conceals the risks.

1. Strongly disagree
2. Disagree
3. Neither agree nor disagree
4. Agree
5. Strongly agree

E8. I check the accuracy of the HRI I come across on the Internet by searching multiple websites again.

1. Strongly disagree
2. Disagree
3. Neither agree nor disagree
4. Agree
5. Strongly agree

E9. I check the accuracy of the HRI I come across on the Internet by consulting a healthcare professional.

* Healthcare professionals: physicians, doctors of Korean medicine, dentists, pharmacists, nurses, nursing assistants, etc.

1. Strongly disagree
2. Disagree
3. Neither agree nor disagree
4. Agree
5. Strongly agree

**The following questions are regarding your experience of communicating “health-related information” you come across on the Internet. Please rate how often you engage in the following activities.**

**“Health-related information” refers to any information related to health, such as information regarding diseases, medication, food, and exercise.**

F1. I press the *recommend*, *do not recommend*, *like*, or *dislike* buttons on HRI posts I come across on the Internet to express my opinion.

1. Strongly disagree
2. Disagree
3. Neither agree nor disagree
4. Agree
5. Strongly agree

F2. I write comments on HRI posts I come across on the Internet to express my opinion.

1. Strongly disagree
2. Disagree
3. Neither agree nor disagree
4. Agree
5. Strongly agree

F3. I forward HRI posts I come across on the Internet to others via messaging apps.

1. Strongly disagree
2. Disagree
3. Neither agree nor disagree
4. Agree
5. Strongly agree

F4. I post texts, images, or videos with HRI on the Internet (e.g., blog, online forum, YouTube, social media).

1. Strongly disagree
2. Disagree
3. Neither agree nor disagree
4. Agree
5. Strongly agree

**The following questions are regarding your behaviors when posting, communicating, or expressing opinions (including likes, recommendations, comments) on “health-related information” obtained from the Internet. Please rate how often you engage in the following activities.**

**“Health-related information” refers to any information related to health, such as information regarding diseases, medication, food, and exercise.**

G1. I check whether the HRI on the internet is accurate when I post, forward, or express opinions (e.g., likes, recommendations, comments) through the internet.

1. Strongly disagree
2. Disagree
3. Neither agree nor disagree
4. Agree
5. Strongly agree

G2. I believe that the HRI that I post, forward, or comment on over the Internet (including likes, recommendations, comments) may affect the health or well-being of others or society.

1. Strongly disagree
2. Disagree
3. Neither agree nor disagree
4. Agree
5. Strongly agree

G3. I check whether the HRI I post, forward, or comment on over the Internet (including likes, recommendations, comments) contains any material that violates the law.

1. Strongly disagree
2. Disagree
3. Neither agree nor disagree
4. Agree
5. Strongly agree

G4. I disclose the source of the HRI on the internet when I post or forward it.

1. Strongly disagree
2. Disagree
3. Neither agree nor disagree
4. Agree
5. Strongly agree

**Demographic Survey**

DQ1. What is your sex?

1. Male
2. Female

DQ2. What is your highest level of education?

1. Elementary school graduate or lower
2. Middle school graduate
3. High school graduate
4. University graduate or higher

DQ3. What is your average monthly household income?

1. Less than 2.5 million KRW per month
2. 2.5 million KRW to less than 3.5 million KRW per month
3. 3.5 million KRW to less than 5 million KRW per month
4. 5 million KRW to less than 7.5 million KRW per month
5. 7.5 million KRW or more per month
