# Supplementary material for: Development and Validation of the Media Health Literacy Scale: Assessment Tool Development Study
Source: J Med Internet Res. 2025 May 5;27:e62884. doi: 10.2196/62884 (PMC12089881; doi:10.2196/62884)
Supplement: Multimedia Appendix 5 [file jmir_v27i1e62884_app5.docx]

Multimedia Appendix 4. Item analysis results.

|  | Mean | Standard Deviation | Skewness | Kurtosis | Item-Total correlations |
| --- | --- | --- | --- | --- | --- |
| Item 1. | 4.28 | 0.690 | -0.545 | -0.356 | .432^**^ |
| Item 2. | 4.31 | 0.706 | -0.683 | -0.094 | .451^**^ |
| Item 3. | 4.32 | 0.711 | -0.676 | -0.266 | .477^**^ |
| Item 4. | 4.26 | 0.687 | -0.453 | -0.536 | .517^**^ |
| Item 5. | 4.20 | 0.680 | -0.389 | -0.371 | .507^**^ |
| Item 6. | 4.02 | 0.677 | -0.337 | 0.158 | .598^**^ |
| Item 7. | 3.71 | 0.811 | -0.142 | -0.493 | .578^**^ |
| Item 8. | 4.10 | 0.641 | -0.320 | 0.282 | .552^**^ |
| Item 9. | 4.02 | 0.656 | -0.145 | -0.258 | .597^**^ |
| Item 10. | 4.04 | 0.670 | -0.243 | -0.119 | .587^**^ |
| Item 11. | 4.03 | 0.668 | -0.276 | 0.275 | .605^**^ |
| Item 12. | 3.84 | 0.693 | -0.107 | -0.262 | .613^**^ |
| Item 13. | 3.94 | 0.622 | -0.211 | 0.301 | .605^**^ |
| Item 14. | 3.90 | 0.694 | -0.195 | -0.155 | .647^**^ |
| Item 15. | 3.85 | 0.732 | -0.259 | 0.024 | .648^**^ |
| Item 16. | 3.81 | 0.766 | -0.170 | -0.253 | .630^**^ |
| Item 17. | 3.80 | 0.693 | -0.217 | -0.027 | .609^**^ |
| Item 18. | 3.75 | 0.770 | -0.235 | -0.267 | .582^**^ |
| Item 19. | 3.77 | 0.703 | -0.274 | 0.227 | .619^**^ |
| Item 20. | 3.83 | 0.707 | -0.156 | -0.016 | .625^**^ |
| Item 21. | 3.58 | 0.831 | -0.092 | -0.435 | .589^**^ |
| Item 22. | 3.44 | 0.918 | -0.286 | -0.083 | .561^**^ |
| Item 23. | 3.26 | 0.968 | -0.217 | -0.451 | .484^**^ |
| Item 24. | 3.39 | 0.907 | -0.246 | -0.226 | .546^**^ |
| Item 25. | 3.20 | 0.981 | -0.121 | -0.756 | .454^**^ |
| Item 26. | 3.62 | 0.781 | -0.221 | -0.048 | .544^**^ |
| Item 27. | 3.77 | 0.670 | -0.303 | 0.427 | .565^**^ |
| Item 28. | 3.54 | 0.820 | -0.127 | -0.389 | .530^**^ |
| Item 29. | 3.62 | 0.792 | -0.204 | 0.015 | .581^**^ |

** p < 0.01
